# The Role of Intergenerational Neighborhood Relations for Precarious Aging During the COVID-19 Pandemic in Germany

**DOI:** 10.1093/geroni/igab046.2035

**Published:** 2021-12-17

**Authors:** Anna Wanka

**Affiliations:** Goethe University Frankfurt, Frankfurt, Hessen, Germany

## Abstract

Throughout the Covid-19 pandemic, the immediate living environment has significantly gained importance - particularly for people framed as ‘risk-groups’, such as older adults. Effects of contact restrictions to contain the spread of the virus have affected inequalities, uncertainties and loneliness in later life differently depending on the intergenerational relations, informal infrastructures of provisioning and networks of solidarity given in a certain neighborhood. The paper presents findings from a recent mixed-methods study in Frankfurt, Germany, combining a quantitative survey (n=1.000) with a longitudinal qualitative study (n=60). Results show how intergenerational neighborhood relations can play a crucial role in mediating risks of pandemic precariousness in later life, but also how older adults themselves significantly contributing to neighborhood networks of provisioning. Strengthening such very local relations is key to protecting all age groups from the effects of crises beyond the pandemic, and, in conclusion, ways to do so are being discussed.

